# Supplementary material for: “Freedom to Breathe”: Youth Participatory Action Research (YPAR) to Investigate Air Pollution Inequities in Richmond, CA
Source: Int J Environ Res Public Health. 2021 Jan 11;18(2):554. doi: 10.3390/ijerph18020554 (PMC7826540; doi:10.3390/ijerph18020554)
Supplement: Supplementary file 1 [file ijerph-18-00554-s001.pdf]

International Journal of
*Environmental Research and Public Health*

MDPI

# Supplemental Materials

**Table S1.** Air pollution and noise pollution measurement results.

| Sample Site | Sampling Dates | NO2 (ppb) | SO2 (ppb) | 1st Spot Noise (dB) | 2nd Spot Noise (dB) | Average Spot Noise (dB) |
|---|---|---|---|---|---|---|
| 1 | 12/21-1/15 | 13.4 | 0.04 | - | 73.9 | 73.9 |
| 2 | 12/21-1/15 | 14.3 | 0.02 | 74.5 | 72.5 | 73.5 |
| 3 | 12/21-1/15 | 14.2 | 0.13 | 85.0 | 71.4 | 78.2 |
| 4 | 12/21-1/15 | 14.9 | 0.07 | 83.0 | 79.9 | 81.5 |
| 5 | 12/21-1/15 | 13.1 | ND | 70.0 | 80.5 | 75.3 |
| 6 | 12/21-1/15 | 12.4 | ND | 81.0 | 75.0 | 78.0 |
| 7 | 12/22-1/15 | 11.1 | 0.03 | 80.0 | 79.8 | 79.9 |
| 8 | 12/22-1/15 | 8.2 | 0.04 | 66.5 | 73.4 | 70.0 |
| 9 | 12/22-1/15 | 10.4 | 0.14 | 71.0 | 67.0 | 69 |
| 10 | 12/22-1/15 | 11.4 | 0.10 | 84.0 | 79.6 | 81.8 |
| 11 | 12/22-1/15 | 10.9 | 0.01 | 68.0 | 65.4 | 66.7 |
| 12 | 12/22-1/15 | 10.8 | 0.06 | 67.0 | 68.8 | 67.9 |
| **Average** | | 12.1 | 0.06 | 75.5 | 73.9 | 74.6 |

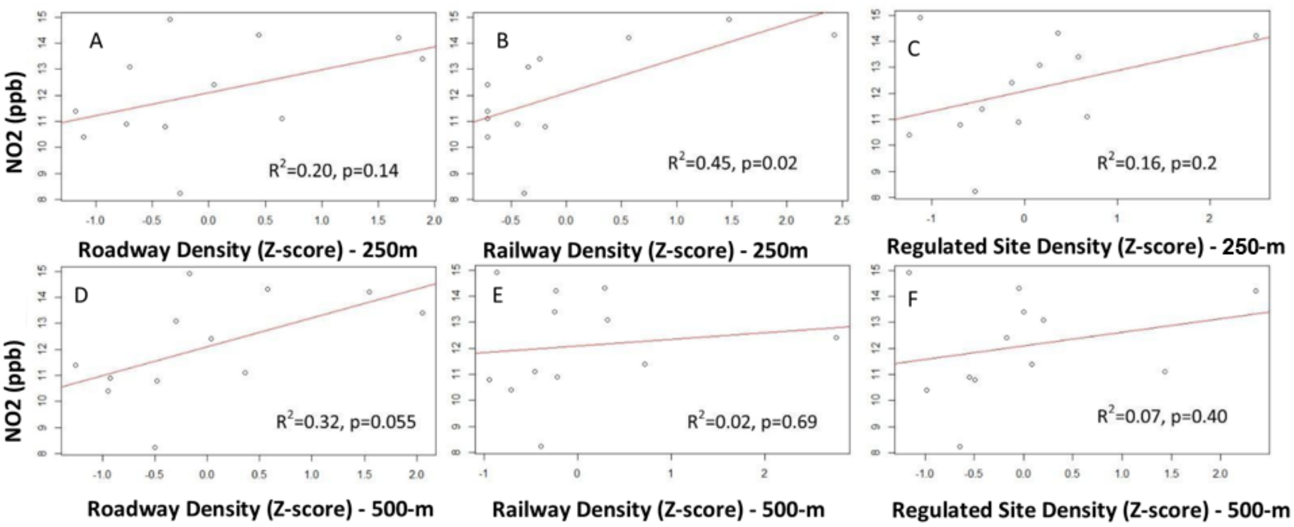


**Figure S1.** Scatter plots displaying the positive relationship between air pollution sources and NO2 (SO2 not shown because there was no evidence of a relationship between sources and SO2 levels): A. Roadway density – 250-m; B. Railway density – 250-m; C. Regulated site density – 250-m; D. Roadway density – 500-m; E. Railway density – 500-m; F. Regulated site density – 500-m.

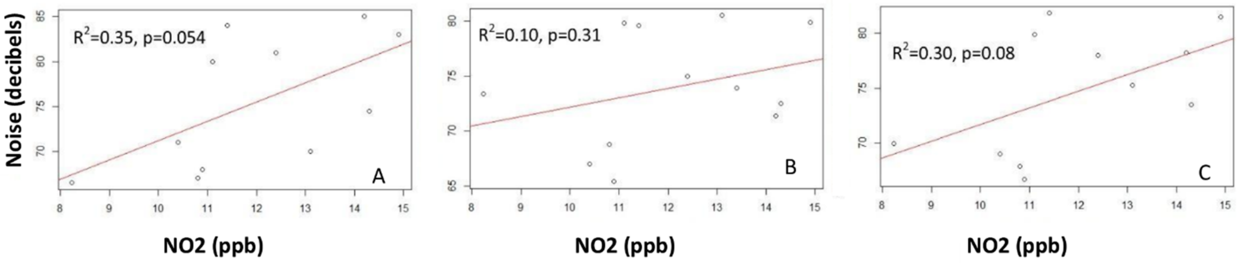


**Figure S2.** Scatter plots showing the positive relationship between NO2 and spot noise measurements (SO2 not shown because there was no evidence for a relationship between noise and SO2). (A) First measurement during sampler deployment; (B) Second measurement during sampler retrieval; (C) Average of both spot noise measurements.

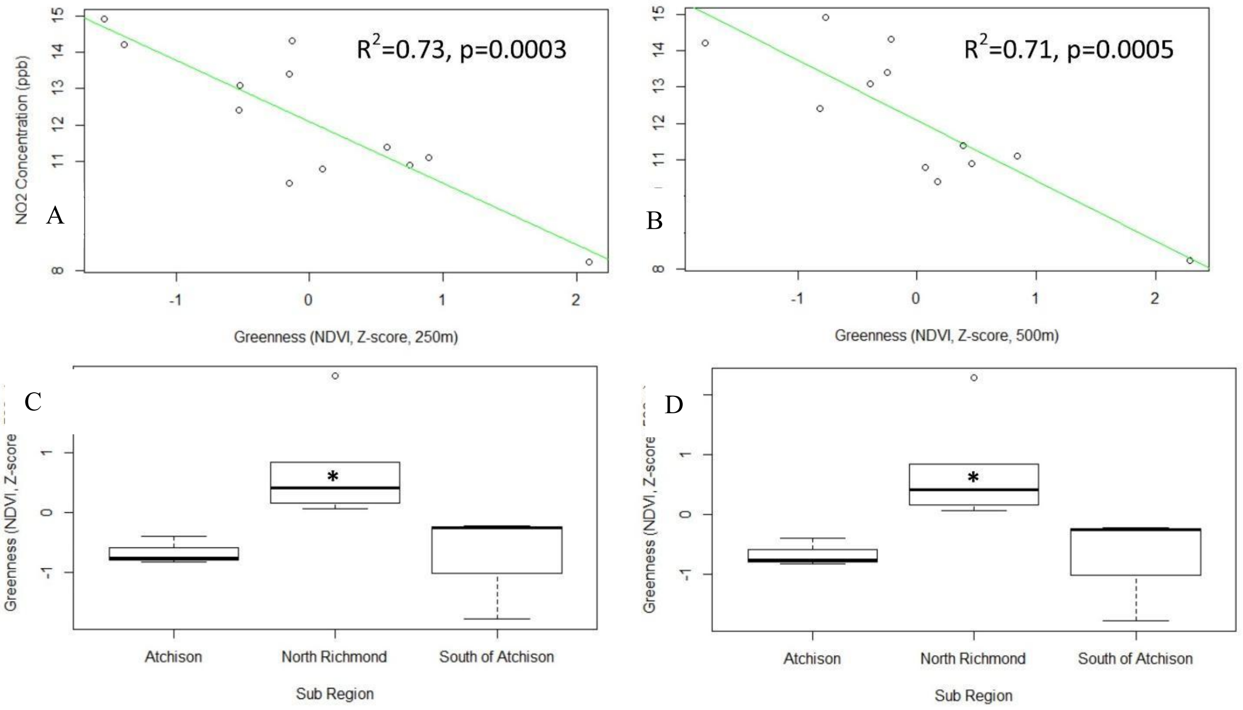


**Figure S3.** The first row (A and B) scatter plots show the negative relationship between area-level greenness (A = 250-m buffer; B = 500-m buffer) and NO2 concentrations (SO2 not shown since there was no evidence for a relationship between greenness and SO2). Second row (C and D) boxplots indicate area-level greenness for the different neighborhoods (C = 250-m buffer; D = 500-m buffer). North Richmond has significantly ($p < 0.05$) higher area-level greenness.
